# Intravascular Large B-Cell Lymphoma

**DOI:** 10.1155/2023/5596890

**Published:** 2023-09-15

**Authors:** Mehdi Loukhnati, Fatima Ezzahra Lahlimi, Illias Tazi

**Affiliations:** Department of Hematology and Bone Marrow Transplantation Mohammed VI University Hospital, Cadi Ayyad University, Marrakesh 40080, Morocco

## Abstract

Intravascular large B-cell lymphoma (IVBCL) is a very rare and aggressive subtype of extranodal diffuse large B-cell lymphoma (DLBCL) involving the growth of lymphoma cells within blood vessels of all organ types. We present the case of a 55-year-old North-African man with no prior history of neoplastic disease presenting with weight loss and an isolated splenomegaly. Investigations led to the diagnosis of this disease. To the best of our knowledge, this is the first case recorded in Africa. Through this article, we discuss this case and outline the common presentation, paraclinical investigations, and treatment options of IVBCL.

## 1. Introduction

Intravascular large B-cell lymphoma (IVBCL) is a very rare subtype of non-Hodgkin B-cell lymphoma. It was first reported by Pleger and Tappeiner in 1959 and characterized by restricted growth of neoplastic cells within the lumina of blood vessels [1]. Its incidence is estimated at 0.095/1 000 000 with an age median of 75 years [2]. The clinical presentation of IVBCL is widely variable making detection very challenging, as most patients present late in the course of the disease.

## 2. Case Presentation

A 55-year-old North-African male with no comorbidities presented in February 2021 with a six-month history of pain on the upper left side of the abdomen and weight loss estimated at 14 kg.

On physical examination, the patient was stable with normal vital signs. There was marked splenomegaly, 8 cm below the costal margin, but no palpable lymphadenopathy, neither hepatomegaly nor skin lesions. Neurological examination did not reveal neurological deficit.

Laboratory investigations showed hemoglobin at 14.6 (11.5–15.5) g/dL, mean corpuscular volume (MCV) at 92 (82−15.5) fL, and platelets at 153 (150–450) G/L. His white cell count (WCC) was normal with lymphocyte count at 1062/mm^3^. Liver enzymes, such as aspartate transaminase (ASAT), alanine transaminase (ALAT), and alkaline phosphatase, were within normal range. The serum lactate dehydrogenase (LDH) was elevated, at 640 (135–225) U/L. The serum creatinine was normal at 0.8 (0.67–1.17) mg/dl. B2 microglobuline was of 3.63 mg/L, and serum protein electrophoresis was negative for any monoclonal gammopathy.

Peripheral blood smear revealed approximately 9% of blasts with increased nucleo-cytoplasmic (N/C) ratio and slightly basophilic cytoplasm without granulations. Nuclear contours were irregular, and chromatin was fine with one or two well-defined nuclei. Two separate lymphocyte populations were observed. The first ones (12%) were small with condensed chromatin and reduced cytoplasm. The second ones (8%) were atypic cells with irregular contoured and notched nucleoli.

**Figure 1:**
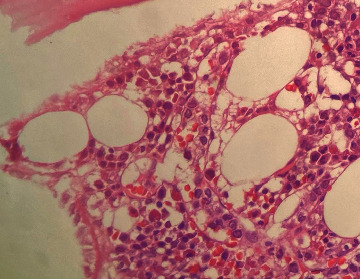
Bone marrow biopsy: palisade arrangement of atypical cells mixed with red blood cells within the vessels.

Bone marrow aspirate smear showed many medium to large cells with round or irregular contoured nucleus. Chromatin was young with increased N/C. No hemophagocytosis was found (Figure 1).

Flow cytometry on medullary aspirate did not find any blastic infiltration and lymphocytic immunophenotyping showed positive expression of CD20, CD19, CD79b, FMC7, CD22, CD25, CD103, CD11c, and CD5 (weak) with intense expression of kappa light chain and negative expression of CD23 and CD43.

Karyotype was normal in 13 mitoses, with addition of mitogene B karyotype found 78–82, X, −Y, +X, +4, +5, +5, del(6) (q22)x2, +6, +7, +11, +14, +15 der(17) t(17; ?) (p11; ?) x2, +17, −19, +20, +21, +3, −6mar [10].

Bone marrow biopsy showed intrasinusoidal pattern with interstitial infiltrate that included large-sized cells with round and irregular nuclei, prominent nucleoli, and reduced cytoplasm. Immunohistochemistry analysis showed positive expression for CD20 and negative expression for CD30 and CD3. The morphology and immunohistochemical profile indicated a diagnosis of intravascular B-cell lymphoma.

**Figure 2:**
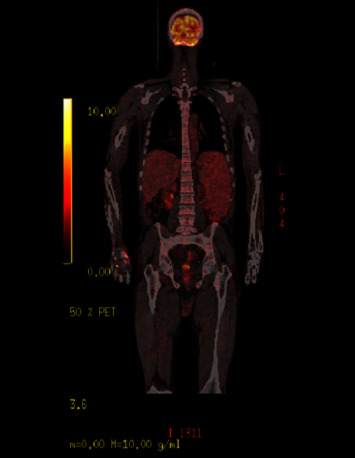
PET scan showing bone marrow and spleen hypermetabolism.

A CT scan of the chest, abdomen, and pelvis was performed. It revealed massive and isolated splenomegaly (23.5 cm diameter). The patient displayed high diffuse uptake of 18F-fluorodeoxyglucose (FDG) in the enlarged spleen and bone marrow (SUV max 3.38) (Figure 2). Cerebral MRI was not performed initially.

Our patient received 6 cycles of R-CHOP (rituximab, cyclophosphamide, doxorubicin, vincristine, and prednisone) and achieved complete metabolic response (DEAUVILLE 1) after four cycles with complete regression of splenomegaly. During the last twelve months of follow-up, no recurrence was noted.

## 3. Discussion

Intravascular large B-cell lymphoma (IVBCL) is a rare subtype of diffuse large B-cell lymphoma characterized by exclusive proliferation of neoplastic cells within small to intermediate-sized blood vessels.

Intravascular large cell lymphomas of natural killer (NK)/T-cell origin exist and are extremely rare [3]. The incidence is estimated at 0.095/1 000 000 with an age median of 75 years and gender predilection of IVBCL are not known. Three major presentations are described: classic variant, cutaneous variant, and hemophagocytic variant [4, 5].

It can precede, follow, or occur simultaneously with non-Hodgkin lymphomas such as large B-cell lymphoma, lymphocytic lymphoma, and follicular lymphoma [6].

Although some theories have been proposed, the mechanism by which IVBCL cells engraft into blood vessels remains largely unknown. Those cells have been demonstrated to lack some molecules such as surface protein CD29 and CD54 which are important for extravasation of lymphocytes and do not express matrix metalloproteinase 2 and 9 critical for parenchymal invasion [7, 8].

Clinical presentation is very heterogeneous, and diagnosis is very difficult with roughly half the cases diagnosed postmortem on random skin and bone marrow biopsy.

In immunohistological examination, IVBCL cells are large lymphoid cells with one or more nucleoli and scant cytoplasm within vessel lumina. They express B-cell-associated antigens: CD20, CD79b, and PAX 5. They are characterized with immunophenotypic heterogeneity of CD10, BCL6, and BCL2 with a nongerminal center pattern in 75 to 80% of cases. MYD88 and CD79b genes' mutation rates are, respectively, 88% and 26%.

Laboratory findings are nonspecific, and anemia is the most frequent cytopenia (2/3 cases) and increased serum levels of LDH and *β*2-microglobulin.

The classical variant is more common in Western countries. The performance status is often altered and B signs including fever, night sweats, and weight loss are often present. It is characterized by clinical heterogeneity, ranging from paucisymptomatic forms to severe forms with higher neurological and cutaneous involvement. In cases of central nervous system (CNS) involvement, most frequently observed symptoms are related to ischemia and infarction. Brain imaging findings are nonspecific, and cerebrospinal fluid (CSF) study often shows hyper proteinorachie with rarely lymphomatous infiltration. The most common differential diagnosis is vasculitis, and 15% of the cases are associated with other solid tumors [4, 6].

The cutaneous variant represents 26% of the cases and is limited to the skin without systemic involvement. It affects almost exclusively younger Caucasian females. The presentation is nonspecific and may involve single or multiple lesions. The prognosis is better than that of the other two variants probably due to the isolated skin involvement and timely diagnosis [4, 6, 9].

Hemophagocytosis-associated IVLBCL, the so called Asian variant, is very aggressive and has the worst prognosis. Patients display hemophagocytic syndrome (HPS) with involvement of the spleen, liver, and bone marrow [5, 6].

The diagnosis of IVBCL is very challenging and is associated with poor prognosis when delayed making timely diagnosis essential. It has been suggested in patients with suspicious IVBCL the usefulness of random skin biopsy despite absence of evident skin involvement [10].

The differential diagnosis of intravascular lymphoma includes hairy cell leukemia (HCL), marginal zone lymphoma (MZL), splenic diffuse red pulp small B-cell lymphoma (SDRPL) and hepatosplenic T-cell lymphoma [6].

Current clinical practice shows that there is no absolutely reliable IVLBCL staging parameter. Ann Arbor Staging System IE disease is present in 40% of patients, and the remaining 60% of patients almost exclusively show stage IV of the disease.

For IVBCL patients, staging work-up should include routine CNS magnetic resonance imaging and bone marrow biopsy, which have dual roles as diagnostic and staging tools. Rare cases (5%) involving peripheral blood are associated with bone marrow infiltration.

There is no agreed consensus for the treatment of IVBCL, due to its rarity and absence of corresponding trials. Studies have shown that anthracycline-based chemotherapy can improve outcomes in patients with IVBCL. The addition of rituximab to chemotherapy further improves outcomes. Thus, R-CHOP is considered the standard treatment for systemic IVBCL. The role of high-dose chemotherapy followed by autologous hematopoietic stem cell transplantation (auto-HSCT) remains a point of debate [6].

## 4. Conclusion

In conclusion, IVBCL is a rare and very aggressive disease with variable presentations and difficulties in timely diagnosis. It should be considered as differential diagnosis even in African patients, and further research remains imperative to better understand this unique non-Hodgkin type of lymphoma.

## Data Availability

The data can be made available from the corresponding author upon reasonable request.
